# Anti-Inflammatory Effects of *Siegesbeckia orientalis* Ethanol Extract in *In Vitro* and *In Vivo* Models

**DOI:** 10.1155/2014/329712

**Published:** 2014-08-26

**Authors:** Yong-Han Hong, Li-Wen Weng, Chi-Chang Chang, Hsia-Fen Hsu, Chao-Ping Wang, Shih-Wei Wang, Jer-Yiing Houng

**Affiliations:** ^1^Department of Nutrition, I-Shou University, Kaohsiung 82445, Taiwan; ^2^Institute of Biotechnology and Chemical Engineering, I-Shou University, Kaohsiung 84001, Taiwan; ^3^Department of Obstetrics and Gynecology, E-DA Hospital, I-Shou University, Kaohsiung 82445, Taiwan; ^4^Division of Cardiology, E-DA Hospital, I-Shou University, Kaohsiung 82445, Taiwan; ^5^Division of Allergy, Immunology, and Rheumatology, Department of Internal Medicine, E-DA Hospital, I-Shou University, Kaohsiung 82445, Taiwan

## Abstract

This study aims to investigate the anti-inflammatory responses and mechanisms of* Siegesbeckia orientalis* ethanol extract (SOE). In cell culture experiments, RAW264.7 cells were pretreated with SOE and stimulated with lipopolysaccharide (LPS) for inflammatory mediators assay. In animal experiments, mice were tube-fed with SOE for 1 week, and s.c. injected with *λ*-carrageenan or i.p. injected with LPS to simulate inflammation. The degree of paw edema was assessed, and cytokine profile in sera and mouse survival were recorded. Data showed that SOE significantly reduced NO, IL-6, and TNF-α production in LPS-stimulated RAW264.7 cells.* In vivo* studies demonstrated that mice supplemented with 32 mg SOE/kg BW/day significantly lowered sera IL-6 level and resulted a higher survival rate compared to the control group (*P* = 0.019). Furthermore, SOE inhibited LPS-induced NF-*κ*B activation by blocking the degradation of I*κ*B-α. The SOE also reduced significantly the phosphorylation of ERK1/2, p38, and JNK in a dose-dependent manner. In summary, the* in vitro* and* in vivo* evidence indicate that SOE can attenuate acute inflammation by inhibiting inflammatory mediators via suppression of MAPKs- and NF-*κ*B-dependent pathways.

## 1. Introduction

Growing evidence suggests that systemic inflammation is associated with increased risk of chronic diseases [1, 2]. The mechanisms of inflammation may involve activation of macrophages and T lymphocytes, as well as the release of proinflammatory mediators, including tumor necrosis factor- (TNF-) α, interleukin- (IL-) 1, IL-6, nitric oxide (NO), and prostaglandin E_2_ (PGE_2_) that amplify the inflammatory activity [3]. Appropriate production of these mediators promotes effective innate immune response; however, excessive inflammation may cause such conditions as chronic inflammation, sepsis, and even death [4].

Notably, mitogen-activated protein kinases (MAPKs) play an important role in the regulation of proinflammatory mediators on cellular responses [5, 6]. Lipopolysaccharide (LPS) stimulation on macrophages can mimic inflammatory responses [7]. The MAPK cascade, the key downstream pathway for LPS-induced signaling events, leads to several functional responses. Activated MAPKs are responsible for phosphorylating and activating numerous transcription factors, including ERK1/2, p38, SAPK/JNK, and NF-*κ*B, which then translocate into the nucleus of cells and induce the transcriptional activation of various inflammatory and immune genes [8]. Beside LPS, sulfated polysaccharide and carrageenan can also act as the proinflammatory agent. Recently, experimental evidence has shown that carrageenan-activated inflammatory cascades are related to generation of reactive oxygen species and may be integrated at the level of I*κ*B kinase (IKK) signalosome, leading to degradation of I*κ*B-α and translocation of NF-*κ*B to nuclei [9]. Accordingly, modulating effectively aberrant production of proinflammatory mediators can reduce inflammatory response. Thus, analyzing the expression of proinflammatory mediators may facilitate identification of anti-inflammatory substances.

The dietary application of natural products, including food materials and Chinese medicinal herbs, has been proposed that may prevent inflammatory diseases [10, 11]. Herba Siegesbeckiae, one of common Chinese medicinal foods, has been used to treat rheumatoid arthritis, malaria, and snakebite based on its ability to dispelling wind, eliminating dampness, and strengthening the sinews [12]. To date, three originals,* Siegesbeckia orientalis* L.* Siegesbeckia pubescens* Makino, and* Siegesbeckia glabrescens* Makino have been identified. Among them,* S. orientalis* has been reported to have antirheumatic [13], antiallergic [14], and immunosuppressive activities [15].* S. orientalis*'s pure component, kirenol, was also found to have a topical application on the attenuation of skin inflammation in murine models [16]. However,* S. orientalis* via oral administration has not been reported regarding its* in vivo* anti-inflammatory activity and related mechanisms. Therefore, this study investigates the anti-inflammatory responses and their related mechanisms in inflammatory cells or mice pretreated with ethanol extract from* S. orientalis* (SOE).

## 2. Materials and Methods

### 2.1. Reagents and Plant Materials

The reagents lipopolysaccharide (LPS,* E. coli* serotype O55:B5), indomethacin (IND), and ammonium pyrrolidinedithiocarbamate (PDTC), obtained from Sigma-Aldrich (St. Louis, MO, USA), were all of analytical grade and dissolved in phosphate buffer saline as a stock. The* S. orientalis* L. samples were purchased from a local herbal store (Yuanshan Company, Kaohsiung, Taiwan). The sample's original was identified and its DNA polymorphism had been reported [17]. In this study, the samples were freeze-dried and then ground into powder. The dried powder (9.3 kg) was extracted with a 5-fold volume of 95% ethanol by stirring at room temperature for 1 day. This step was repeated three times. The extracted solutions were collected and filtered through filter paper (Whatman number 1; Whatman Paper Ltd., Maidstone, Kent, UK). The SOE was acquired by removing solvent via a rotary evaporator and dried in a freeze dryer. The dry weight of this extract was 489 g, and the yield was 5.3%. The SOE was dissolved in dimethyl sulfoxide (DMSO; the final DMSO concentration never exceeded 0.1% in medium) for cell culture test and was dissolved in sunflower oil for tube feeding in the mice experiment.

### 2.2. Cell Culture

The RAW264.7 cells (Bioresource Collection and Research Center; Hsinchu, Taiwan) were cultured in Dulbecco's modified Eagle's medium (DMEM-F12) supplemented with 10% inactivated fetal bovine serum, 0.01% glutamine, 1% penicillin/streptomycin, 0.02% sodium bicarbonate, and pH 7.2–7.4. The cells were cultivated in a humidified incubator at 37°C with 5% CO_2_ and 95% air. In this experiment, the RAW264.7 cells were seeded on 6 cm dishes at a cell density of 7 × 10^5^ cells/mL for Western blotting assay, or on 96-well plates at a cell density of 5 × 10^4^ cells/well for culture medium test. The cells were then pretreated with various concentrations of SOE for 1 h before adding 1 *μ*g/mL LPS for the indicated assay. In the Western blotting assay, the cell lysate was collected for detection of target proteins after 1 h (MAP kinase family) or 12 h (iNOS) stimulation. In the culture medium test, culture supernatants were harvested for analysis of the production of proinflammatory mediators after stimulation for 48 h. The cell viability was evaluated using the MTT method. The medium solution was removed after cultivation. An aliquot of 100 *μ*L of DMEM medium containing 1 mg/mL of 3-(4,5-dimethylthiazol-2-yl)-2,5-diphenyltetrazolium bromide (MTT) was loaded to the plate. The cells were incubated for 3 h, and then the medium solution was removed. An aliquot of 100 *μ*L of DMSO was added to the plate, which was shaken until the crystals dissolved. The cytotoxicity against cells was determined by measuring the absorbance of the converted dye at 570 nm in an ELISA reader (Model 550, Bio-Rad Laboratories, Hercules, CA, USA).

### 2.3. NO Determination

Griess reagent was freshly prepared from reagents A (1% sulfanilamide in 2.5% phosphoric acid) and B (0.1% N-1-naphthylethylenediamide dihydrochloride in water) at a ratio of 1 : 1. An equal volume of Griess reagent was added to supernatants from cells treated with test samples in a 96-well plate for 10 min. Absorbance was measured by an ELISA reader at 540 nm. The NO concentrations were determined using a NaNO_2_ standard curve.

### 2.4. Cytokine Production Assay

Production of cytokines TNF-α and IL-6 in cell supernatants and mice serum was assayed using a commercial ELISA kit (eBioscience, Minneapolis, MN, USA). Briefly, primary anti-IL-6 or TNF-α antibodies were coated onto 96-well plates. After overnight incubation, plate wells were washed with washing buffer and blocked with blocking solution for 1 h. After washing, diluted supernatants or sera were added to wells for 2 h incubation. Next, wells were washed with washing buffer and biotin-conjugated anti-IL-6 or TNF-α antibody was added for 1 h. The wells were then washed and horseradish peroxidase-conjugated streptavidin was added for 30 min, washed, and incubated with tetramethylbenzidine (TMB; Clinical Science Products, Mansfield, MA, USA). Absorbance was measured by an ELISA reader at 620 nm. Data were calculated according to standard curves of cytokines.

### 2.5. Western Blot Analysis

The cells were trypsinized, washed twice with phosphate buffered saline (PBS), and lysed with lysis buffer (modified RIPA buffer) at 4°C. The pellet cellular debris was removed by centrifugation at 12500 rpm for 30 min and the supernatants were then either analyzed immediately or stored at −80°C. Protein concentrations were measured by BCA Protein Assay Kit (Pierce, Rockford, IL, USA). Lysates in sample buffer (2% SDS, 10% glycerol, 80 mM Tris-base, 720 mM* DL*-dithiothreitol, and 0.001% bromophenol blue) were denatured at 95°C for 5 min. Equivalent amounts of protein (25 *μ*g) from total cell lysates were subjected to SDS-polyacrylamide gel electrophoresis (PAGE) and the proteins were transferred onto polyvinylidene difluoride (PVDF) membrane (Millipore, Temecula, CA, USA). Nonspecific binding was blocked by soaking the membrane in Tris-buffered saline (TBS, 20 mM Tris-base, and 300 mM NaCl) containing 5% fat-free milk for 1 h. The membrane was incubated with primary antibodies (anti-p38, anti-ASPK/JNK, anti-ERK, anti-I*κ*B-α and anti-NF-*κ*B at 1 : 5000 in TBS; anti-actin at 1 : 7500 in TBS) (Cell Signaling Technology, Danvers, MA, USA) overnight at 4°C. The membrane was then incubated with a secondary antibody, a goat antirabbit IgG, or goat antimouse IgG conjugated to horseradish peroxidase. The protein levels were determined by using enhanced chemiluminescence (ECL) plus western blotting detection reagents (Amersham Bioscience, Uppsala, Sweden) and the bands intensities were scanned. Densitometric analyses were conducted using the Quantity One software (Bio-Rad). Incubation with polyclonal mouse antihuman *β*-actin antibody was performed for comparative control.

### 2.6. Experimental Animals

Six-week-old female BALB/c mice and 7-week-old female ICR mice were purchased from the National Animal Center (Taipei, Taiwan). These mice were maintained in an air-conditioned room at 23 ± 2°C on a regulated 12 h light-dark cycle. They were fed a nonpurified diet (Lab Rodent Chow 5001, Ralston Purina Inc., St. Louis, MO, USA) for adaptation. At age of 9 weeks, the mice were started on dietary treatment. Animal care and handling conformed to the National Institutes of Health's Guide for the Care and Use of Laboratory Animals [18].

### 2.7. SOE Treatment Prior to *λ*-Carrageenan-Induced Paw Edema

To identify the effects of the SOE on local acute inflammation, 28 9-week-old ICR mice were divided randomly into four groups: the control group (*n* = 8), the LSOE group (*n* = 8), the HSOE group (*n* = 8), and the IND group (*n* = 4, positive control). The control and the IND groups were tube-fed daily with 100 *μ*L sunflower oil, while the LSOE and the HSOE groups were tube-fed daily with 10 and 32 mg SOE/kg BW in 100 *μ*L sunflower oil, respectively. All mice also had free access to chow diet and water. These oral doses of SOE were derived from the effective dose of 50 *μ*g/mL in RAW264.7 cells and primary macrophages based on a previous report [19]. After tube feeding with either sunflower oil or SOE for 1 week, 50 *μ*L of 2% *λ*-carrageenan (in saline) and 50 *μ*L saline was injected subcutaneously (s.c.) into the right paw and left paw plantar of each mouse, respectively. Mice in the IND group were s.c. injected with 20 mg indomethacin/kg BW 3 h before *λ*-carrageenan challenge. After each *λ*-carrageenan injection, paw volume was measured at 1-hour intervals using a plethysmometer (Apelex 7150, Massy, France). The stimulation index (S.I.) was used to express the degree of murine paw edema, which was calculated as

(1) $$\begin{array}{c} \text{S}.\text{I}.=\frac{\text{the}\;\text{volume}\;\text{of}\;\text{right}\;\text{paw}\;\left(\text{carageenan}\;\text{injection}\right)}{\text{the}\;\text{volume}\;\text{of}\;\text{left}\;\text{paw}\;\left(\text{PBS}\;\text{injection}\right)}. \end{array}$$

### 2.8. SOE Treatment Prior to LPS-Induced Systemic Inflammation

Forty-five 9-week-old BALB/c mice were divided randomly into four groups: the control group (*n* = 13), the LSOE group (*n* = 12), the HSOE group (*n* = 12), and the PDTC group (*n* = 8, positive control). All mice were fed chow diet and supplemented with 100 *μ*L sunflower oil daily. Their experimental doses and treatment durations were the same as those in the *λ*-carrageenan experiment. After 1 week of tube-feeding, all mice were injected intraperitoneally (i.p.) with 15 mg LPS/kg BW to induce systemic inflammation. Mice in the PDTC group were i.p. injected with 50 mg PDTC/kg BW, a dose with anti-inflammatory effects, 1 h before LPS challenge. Sera were collected at 2 and 9 h after LPS challenge for cytokine assay. The life spans of all mice were also recorded.

### 2.9. Gas Chromatography-Mass Spectrometry

GC-MS analysis was performed using Varian 450-GC and 240-MS system (Varian, Salt Lake City, UT, USA) with the electron impact mode (70 eV) injector and a Varian data system. The GC column was VF-5 ms capillary column (30 m × 0.25 mm, film thickness 0.25 *μ*m, FactorFour, USA). Injector and detector temperatures were set at 250°C and 290°C, respectively. Oven temperature was kept at 50°C for 5 min, then raised to 120°C by a rate of 5°C/min, kept at 120°C for 8 min, and then raised to 300°C by a rate of 10°C/min. The carrier gas was helium at a flow rate of 1 mL/min. Diluted samples of 1.0 *μ*L were injected manually and in the splitless mode. The percentages of the compounds were calculated by the area normalization method. The components were identified by comparison of their mass spectra with the NIST MS 2.0 database (Gaithersburg, MD, USA). Caryophyllene oxide, hexadecanoic acid ethyl ester, and caryophyllene were purchased from Sigma-Aldrich. The compound 6,10,14-trimethyl-2-pentadecanone was purchased from Apollo Scientific Co. (Stockport, Cheshire, UK).

### 2.10. High Performance Liquid Chromatography (HPLC) Analysis for Kirenol

The content of kirenol was determined by HPLC (LC-20AT, Shimadzu, Tokyo, Japan). The sample was dissolved in methanol and filtered with a 0.22 mm filter. The diluted sample was analyzed by an Ascentis C18 column (number 581325-U, 5 mm, 250 × 4.6 mm; Supelco, Bellefonte, PA, USA). The mobile phase consisted of acetonitrile/methanol (90 : 10, v/v) and water with a linear gradient elution, 0–25 min for 20–60% acetonitrile/methanol (90 : 10, v/v) and 25–50 min for 60–20% acetonitrile/methanol (90 : 10, v/v) at a flow rate of 1.0 mL/min. The sample injection size was 10 *μ*L. The detection was carried out at 215 nm. The residence time of kirenol was 15.4 min.

### 2.11. Statistical Analysis

Each experiment was performed at least three times. The data are expressed as the means ± SD. The significant difference compared to the control group was statistically analyzed by Student's* t*-test using the SAS software program (SAS/STATA version 8.2; SAS Institute, Cary, NC, USA). Statistical comparison between different survival curves was analyzed by Cox's proportional hazards regression test (STATA version 9.0; Stata Corp., TX, USA). The relationship was analyzed by the simple correlation of the SAS program. Statistical significance is expressed as *P* < 0.05.

## 3. Results

### 3.1. *In Vitro* Anti-Inflammatory Effects of SOE

**Figure 1:**
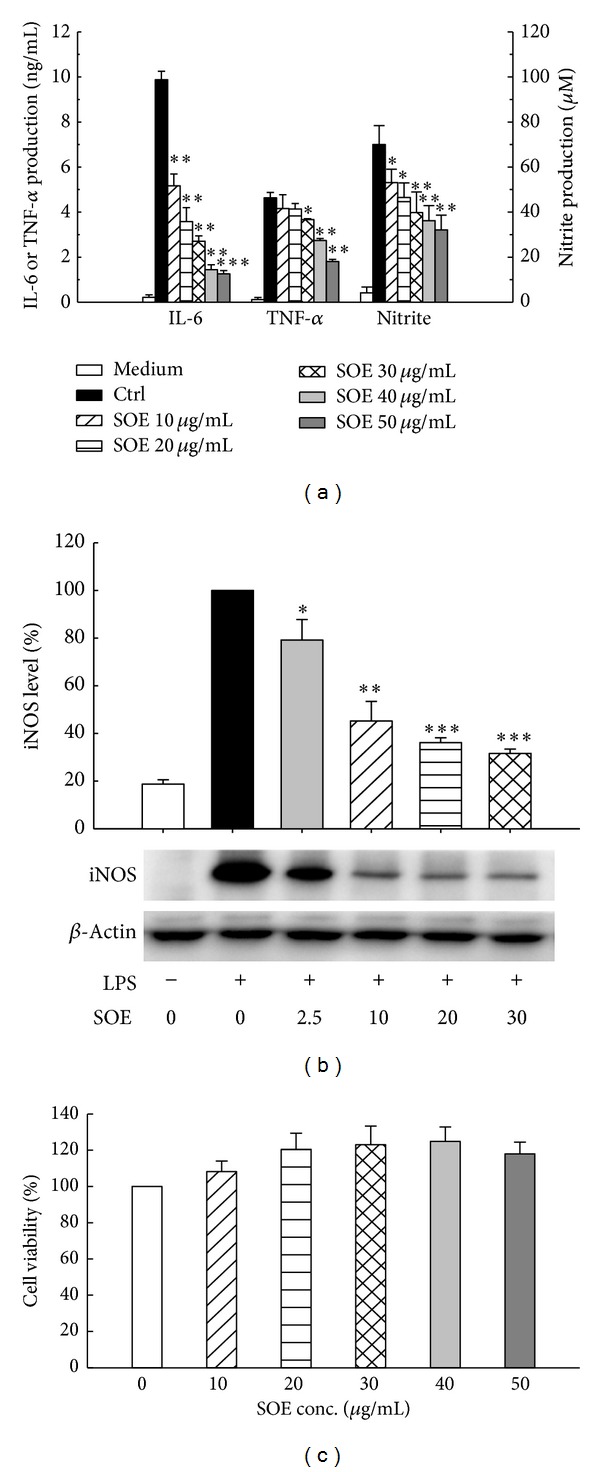
Effects of SOE on proinflammatory mediators production, iNOS expression, and cell viability in LPS-stimulated RAW264.7 macrophages. Cells were pretreated with 0 (control) or the indicated concentration of SOE for 1 h and then stimulated with 1 *μ*g/mL LPS for 12 h (iNOS) or 24 h (proinflammatory mediators). The negative control (medium) is that cells were cultured with medium for the indicated time. (a) Production of IL-6 and TNF-α was measured by an ELISA kit. The nitrite concentration was analyzed using Griess reagent. (b) The expression of the iNOS protein was determined by Western blotting analysis. The iNOS level was quantified by densitometric analysis using the Quantity One software (Bio-Rad). (c) Cell viability was determined by MTT assay. Bar values are means ± SD of three independent experiments in these assays. A significant difference from the control (LPS alone) was indicated as *P < 0.05, **P < 0.01, or ***P < 0.001 by Student's *t*-test.

Notably, NO is endogenously synthesized by inducible nitric oxide synthase (iNOS) through activated NF-*κ*B and MAPK and is strongly related to inflammatory responses. Because formation of NO can induce inflammation, this study first determined whether SOE suppresses NO generation in LPS-stimulated RAW264.7 cells. As presented in Figure 1(a), SOE suppressed NO production dose-dependently. Compared to the Control, NO production was inhibited by 57 ± 7% at the SOE concentration of 50 *μ*g/mL. Next, the inhibitory effects of SOE on the production of proinflammatory cytokines were examined. Experimental data shown in Figure 1(a) indicate that LPS-stimulated IL-6 production was inhibited markedly by SOE pretreatment in a dose-dependent fashion. Consistent with the IL-6 result, SOE significantly inhibited the production of TNF-α dose-dependently. The SOE significantly reduced iNOS protein expression (Figure 1(b)). These data indicate that the inhibitory effect of SOE on NO production is related to its suppression on iNOS protein expression. The cytotoxicity of SOE on LPS-induced RAW264.7 cells was also assessed using the MTT assay. Cell viability did not decrease after incubation for 48 h with SOE up to 50 *μ*g/mL, indicating that SOE is not cytotoxic to cells within this concentration range (Figure 1(c)).

### 3.2. *In Vivo* Effects of SOE on Inflammatory Conditions

**Figure 2:**
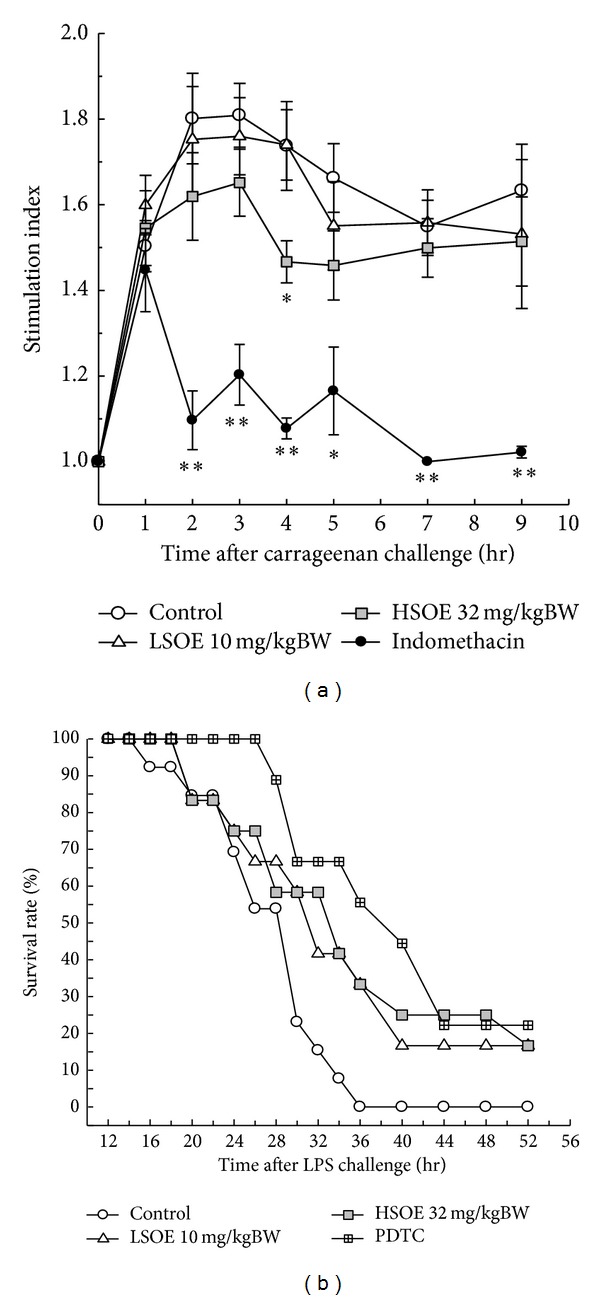
Effects of SOE pretreatment on *λ*-carrageenan-induced paw edema and mouse survival in LPS-challenge model. (a) The ICR mice tube-fed without (control or IND) or with the indicated dose of SOE (10 or 32 mg/kg BW/day) for 7 days were s.c. injected with *λ*-carrageenan into right paw plantar, and the degree of mice paw edema was recorded. The positive control mice were s.c. injected with indomethacin (20 mg/kg BW) at 3 h before *λ*-carrageenan challenge. S.I. = the volume of right paw/the volume of left paw. A significant difference from the control was indicated as *P < 0.05 or **P < 0.01 by Student's *t*-test. (b) The BALB/c mice tube-fed without (control or PDTC) or with the indicated dose of SOE (10 or 32 mg/kg BW/day) for 7 days were i.p. injected with LPS, and mouse survival was recorded. The positive control mice were i.p. injected with PDTC (50 mg/kg BW) at 1 h before LPS challenge. The LSOE, HSOE, and PDTC positive control groups had increased survival using the COX proportion hazards regression test (*P* = 0.027, 0.019, and 0.006, resp.).

Animal experiments were conducted to determine whether 1-week gavage of SOE at the indicated dose ameliorates *λ*-carrageenan-induced and LPS-stimulated inflammation. Experimental results shown in Figure 2(a) indicate that the high dose of SOE (HSOE, 32 mg/kg BW/day) reduced the degree of paw edema at 4 h after *λ*-carrageenan challenge. For systemic inflammation, only half of the control group mice survived at 26 h after LPS challenge, but approximately 75% of mice in the HSOE group were alive at the same time point. At 36 h after LPS challenge, no mouse in the control group survived, but around 30% of those in the HSOE group survived (Figure 2(b)). The survival rate of the HSOE group was significantly higher than that of the control group according to the COX proportional hazards regression test (*P* = 0.019).

Our previous studies examined sera cytokine profile of BALB/c mice with 15 mg/kg BW LPS challenge [19, 20], which is a similar animal model to this investigation. In those studies, the life span was found to be negatively correlated with sera IL-6 and TNF-α level at the early stage (2 h) and the late stage (9 h) of the acute-inflammation period. Therefore, this study examined the level of IL-6 and TNF-α at 2 h and 9 h after LPS challenge. The HSOE group had significantly lower serum IL-6 levels at 2 and 9 h after LPS challenge (Table 1). The PDTC group, a positive control group, had significant lower levels of cytokines than the control group.

### 3.3. *In Vitro* Effects of SOE on NF-*κ*B Activation and MAPK Phosphorylation

**Figure 3:**
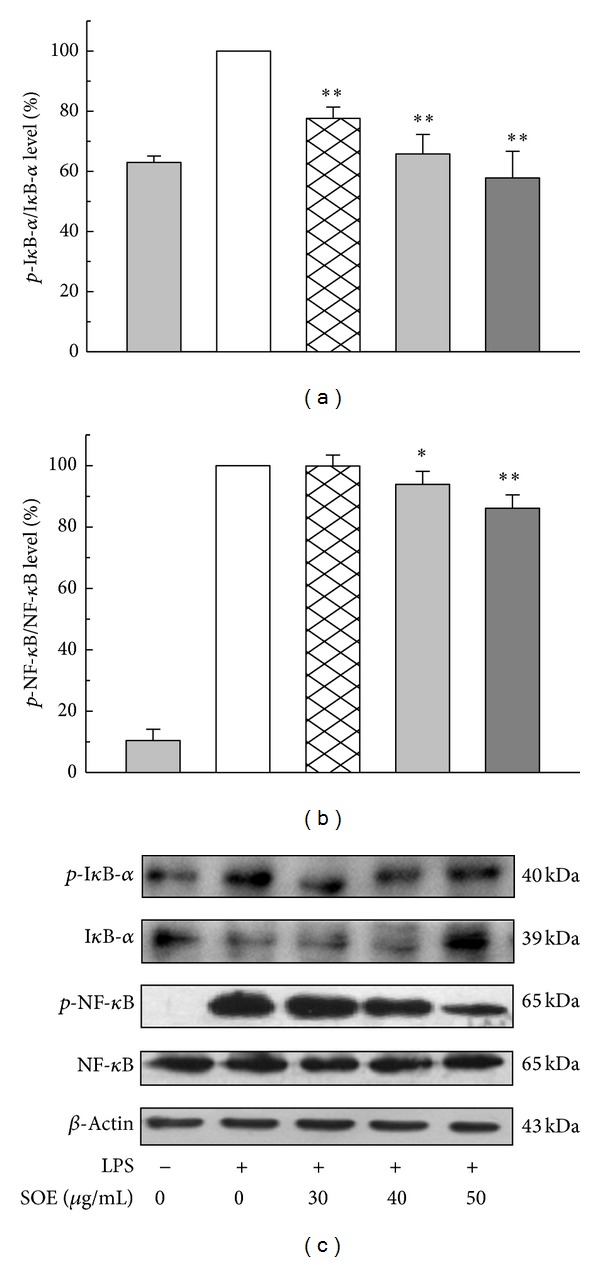
Effects of SOE on the expression of* p*-I*κ*B-α and* p*-NF-*κ*B in LPS-stimulated RAW264.7 cells. Cells were pretreated with the indicated doses of SOE for 1 h and then stimulated with 1 *μ*g/mL LPS for 1 h. Bar values are means ± SD of three independent experiments. The electrophoresis experiment was repeated three times, and one representative result is shown here. A significant difference from the control (LPS alone) was indicated as *P < 0.05 or **P < 0.01 by Student's *t*-test.

**Figure 4:**
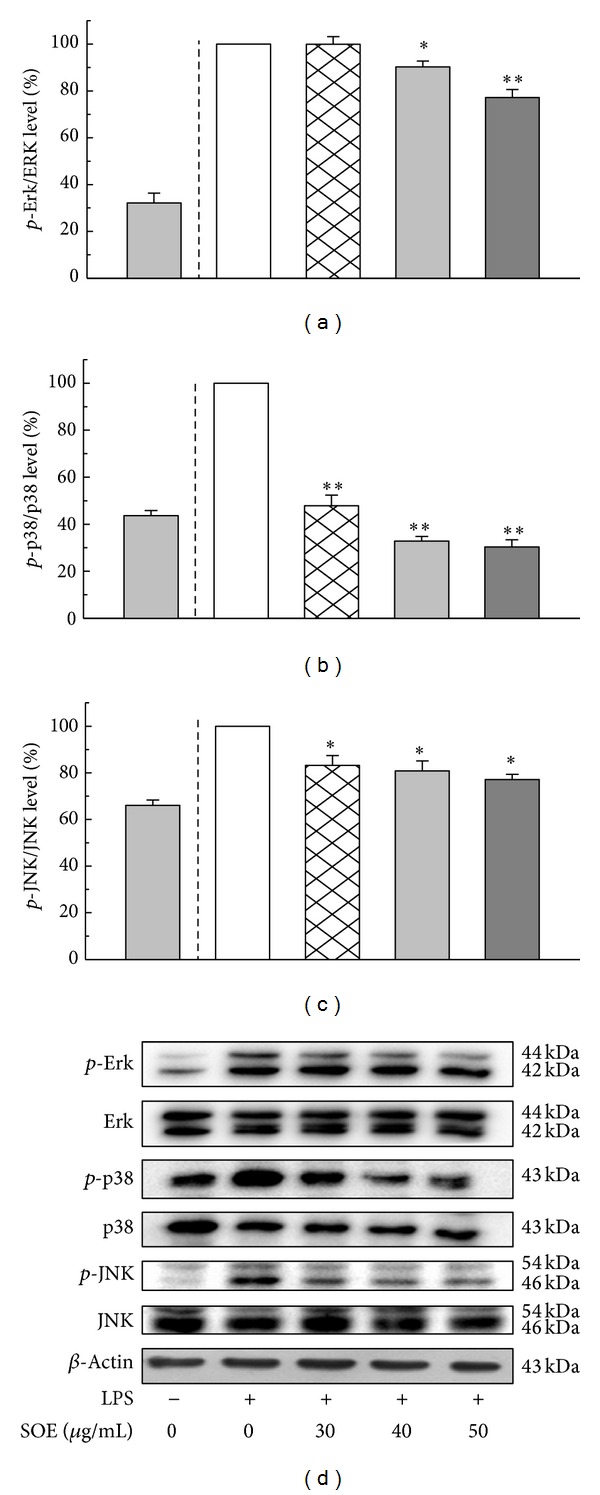
Effects of SOE on the expression of* p*-Erk,* p*-p38, and* p*-JNK in LPS-stimulated RAW264.7 cells. Cells were pretreated with the indicated doses of SOE for 1 h and then stimulated with 1 *μ*g/mL LPS for 1 h. Bar values are means ± SD of three independent experiments. The electrophoresis experiment was repeated three times, and one representative result is shown here. A significant difference from the control (LPS alone) was indicated as *P < 0.05 or **P < 0.01 by Student's *t*-test.

As NF-*κ*B pathway is closely related to the expression of iNOS and proinflammatory cytokines, modulation of SOE on NF-*κ*B activation was examined.  Figure 3 shows Western blot results for LPS-induced RAW264.7 cells under treatment with different SOE concentrations. The SOE significantly suppressed phosphorylation of I*κ*B-α and induced I*κ*B-α expression in a dose-dependent manner. Consistently, SOE reduced the degree of phosphorylation of NF-*κ*B when treatment dose increased. These analytical results suggest that SOE inhibits LPS-induced NF-*κ*B activation by blocking the degradation of I*κ*B-α. To further investigate whether inhibition of NF-*κ*B activation and inflammatory mediators by SOE is modulated by the MAPK pathway, the effects of SOE on LPS-induced phosphorylation of ERK1/2, p38, and JNK were examined. The SOE significantly reduced phosphorylation of ERK1/2, p38, and JNK in a dose-dependent fashion (Figure 4).

### 3.4. Chemical Compositions of SOE

**Figure 5:**
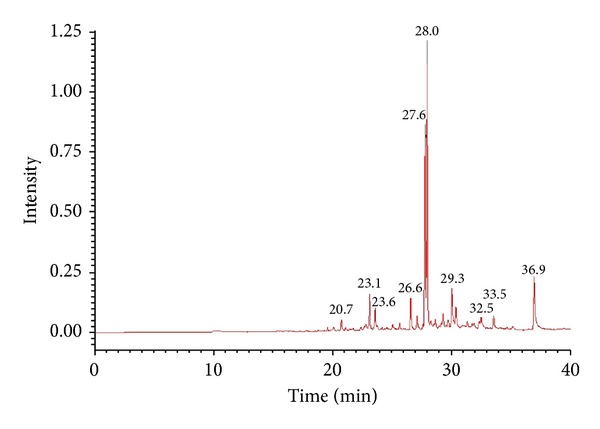
Gas chromatography-mass spectrometry profile of SOE.

The GC-MS analytical results show that at least 20 compounds exist in SOE (Figure 5), of which a total of 10 constituents were identified using mass spectrometry (Table 2). The mass spectra of these compounds were matched with those found in the NIST spectral database. The major compounds in SOE were quantified as caryophyllene oxide (46.9%), [−]-spathulenol (25.7%), and hexadecanoic acid ethyl ester (9.6%) based on the results obtained from GC-MS analysis. Caryophyllene oxide, hexadecanoic acid ethyl ester, caryophyllene, and 6,10,14-trimethyl-2-pentadecanone were confirmed by comparing their mass spectral data with the NIST mass spectral library and commercially available products. Inhibition of NO production in LPS-induced RAW264.7 cells shows that the anti-inflammatory effects of these four compounds were insignificant (data not shown). Therefore, these compounds were not the bioactive anti-inflammatory ingredients of SOE.

The content of kirenol, an ingredient isolated from ethanolic extract of* S. orientalis* and was demonstrated to exhibit significant anti-inflammatory activity [16], in SOE was determined by HPLC analysis. By matching the retention time (RT = 15.4 min) with authentic standards, good linearity was obtained (*R* ^2^ = 0.9995) and the quantity of kirenol was determined as 4.2 ± 0.08 mg/g SOE.

## 4. Discussion

Inflammation, a complex process, is regulated by various immune cells and effector molecules, such as NO and proinflammatory cytokines. Inhibition of these mediators with pharmacological modulators has been proved as an effective therapeutic strategy for reducing inflammatory reactions and risk of inflammatory diseases [21–23]. Macrophages are crucial to host-defense against infections and in inflammation processes through the release of molecules such as NO, PGE_2_, TNF-α, and IL-6. Overproduction of these mediators has been implicated in several inflammatory diseases and cancer [24]. Thus, inhibition of activation of these cells appears to be an important target when treating inflammatory diseases. Stimulation of macrophages with LPS induces high production of NO by iNOS and PGE_2_ by cyclooxygenase- (COX-) 2 [25]. Therefore, a reagent that prevents the release of these mediators or downregulates iNOS or COX-2 expression may possess anti-inflammatory activities.

*S. orientalis*, the most common used original of Herba Siegesbeckiae, and its ethanol extract (SOE), which mimics formulas in medicinal foods, were used to explore its preventive effects against inflammation. In cell culture test, LPS induced-cellular production of IL-6, TNF-α, and NO via iNOS activity was dose-dependently reduced by the SOE in RAW264.7 macrophages (Figure 1). However, decreased PGE_2_ production and COX-2 expression were insignificant within the tested SOE concentration range (data not shown). Moreover, the cell viability rose slightly as the SOE dose increased (Figure 1(c)), implying that SOE exhibited significant anti-inflammatory activity without causing cytotoxicity. These results imply that SOE has potential as an anti-inflammatory agent. With this* in vitro* inhibitory effect of SOE on the production of proinflammatory mediators, the* in vivo *anti-inflammatory potential of SOE was then evaluated.

First, a *λ*-carrageenan-induced paw edema model, which is considered as a highly sensitive tool for evaluating the efficacy of acute inflammation [26], was adopted to assess the preventive effect of SOE on local acute inflammation. It has also been reported that this paw edema would be ameliorated by reducing the levels of IL-1, IL-6, TNF-α, and NO [27]. In this study, SOE at a dose of 32 mg/kg BW/day (HSOE) suppressed significantly *λ*-carrageenan-induced paw edema (Figure 2(a)). Second, a LPS-challenge model, which mimics systemic endotoxemia, was applied to determine whether SOE pretreatment could reduce systemic chronic inflammation. Previous studies had indicated that reduction of sera TNF-α and/or IL-6 levels in LPS-challenged mice could benefit their survival [19, 20]. Our data show that pretreatment with HSOE significantly decreased the sera IL-6 level in mice but did not significantly reduce the level of sera TNF-α (Table 1). However, the HSOE mice had a higher survival rate than the control mice (Figure 2(b)), implying that reduction of IL-6 only remains useful in increasing mice survival in LPS-induced systemic inflammation. These data suggest that the oral administration of SOE has favorable effects on prevention of local and systemic acute inflammation by downregulating production of inflammatory mediators.

The RAW264.7 macrophage cell line has been used as a rapid* in vitro* screening method when studying anti-inflammatory agents [7, 28, 29]. The cytokine profile in the cell model in this study was resembled that in the LPS-induced* in vivo* model. The SOE has similar significant effects on reduction of IL-6 expression in both* in vitro* and* in vivo* studies. However, the effect of SOE on the reduction of the TNF-α level in cell cultures was significant but insignificant in mouse sera. This difference is likely due to the SOE dose used in the cell-model experiment and animal-model experiment. The high dose of SOE of 50 *μ*g/mL in the cell-model test inhibited 84% of IL-6 production and 58% of TNF-α production (Figure 1). In comparison, the high dose of SOE of 32 mg/kg BW/day in the animal-model test inhibited 46% of IL-6 formation and 17% of TNF-α formation (Table 1). Therefore, a higher dose of SOE in the animal-model test would be worthy of further work.

Notably, NF-*κ*B plays an important role in the regulation of cell survival genes and induction of the expression of inflammatory enzymes and cytokines. Therefore, blocking the NF-*κ*B transcriptional activity in the nuclei of macrophages may reduce the expression of iNOS, COX-2, and proinflammatory cytokines and has been considered to be an effective therapy for treating inflammation-related diseases [30]. Under unstimulated conditions, NF-*κ*B is an inactive complex bound to I*κ*Bα in cytosol. After stimulation with LPS, NF-*κ*B is activated through phosphorylation and degradation of I*κ*Bα by increasing IKK or Akt kinase activity [31]. Phosphorylation of NF-*κ*B, regulated by MAPK pathway, plays a vital role in modulating transcriptional activity of NF-*κ*B and is independent of I*κ*Bα proteins [32]. In this study, Western blotting results show that SOE decreased phosphorylated NF-*κ*B level but unaffected total NF-*κ*B level in LPS-stimulated RAW264.7 cells. It was also observed that SOE dose-dependently decreased* p*-I*κ*Bα/I*κ*Bα and SOE at 50 *μ*g/mL significantly increased I*κ*Bα level (Figure 3). These suggest that regulatory effects of SOE on NF-*κ*B activation are partly through modulating the degradation of I*κ*Bα.

The MAPKs are a family of serine/threonine kinases that are involved in a variety of cellular processes. Three MAPK molecules, ERK, p38, and JNK, are activated in response to certain extracellular stimuli such as LPS or carrageenan challenge. These kinases have different downstream targets and mediate diverse cellular responses, including regulation of apoptosis, proliferation, and inflammation [33]. This study shows that treatment by SOE significantly inhibited LPS-induced ERK1/2, p38, and JNK phosphorylation in LPS-stimulated macrophages (Figure 4), which may contribute to the inhibitory effect of SOE on the production of proinflammatory mediators in LPS-induced macrophages.

In GC-MS analysis, 10 SOE constituents were identified using mass spectrometry (Table 2). Among them, 4 compounds, caryophyllene oxide, hexadecanoic acid ethyl ester, caryophyllene, and 6,10,14-trimethyl-2-pentadecanone, were obtained from commercial sources. However, none of these 4 compounds had a significant inhibitory effect on NO production in LPS-induced RAW264.7 cells. Our laboratory has separated SOE into several fractions using a partition procedure with* n*-hexane, ethyl acetate (EA), and methanol. The IC_50_ values of the SOE,* n*-hexane fraction, and EA fraction on the inhibition of NO production in LPS-activated macrophages were 41.8, 53.7, and 5.3 g/mL, respectively, while the methanol fraction was insignificant. Due to the effectiveness of the EA fraction, the bioactive components from this fraction will be screened and separated in our next study.

## 5. Conclusions

In summary,* in vivo* evidence suggests that SOE has significant inhibitory effects on local and systemic acute inflammation, while* in vitro* data reveal that SOE could block activation of NF-*κ*B and MAPKs, thereby inhibiting the induction of iNOS expression and the release of inflammatory cytokines. Taken together, this study demonstrates that SOE is a medicinal food material capable of preventing inflammation. Further studies are still needed to evaluate the detailed molecular mechanisms and define the main bioactive phytochemicals in the SOE.

## Supplementary Material

The Siegesbeckia orientalis L. samples were purchased from Yuanshan Company. The sample's original and its DNA polymorphism had been identified. The samples were extracted with 95% ethanol into the extracts (SOE). The reagents lipopolysaccharide and *λ*-carrageenan were for inflammation induction; Indomethacin and ammonium pyrrolidinedithiocarbamate were as anti-inflammatory reagents.

## Figures and Tables

**Table 1:** Effects of SOE pretreatment on sera cytokine production in LPS-challenged mice^a^.

Group	IL-6 (ng/mL)	TNF-*α* (ng/mL)
At 2 h after LPS challenge		
Control	318 ± 102	2.99 ± 1.52
LSOE	257 ± 89.5	4.32 ± 2.62
HSOE	205 ± 102*	3.51 ± 2.04
PDTC	202 ± 117*	1.47 ± 0.32*
At 9 h after LPS challenge		
Control	151 ± 125	0.63 ± 0.50
LSOE	139 ± 88.7	0.56 ± 0.26^#^
HSOE	81.3 ± 46.5*	0.52 ± 0.35^#^
PDTC	27.9 ± 15.4**	0.25 ± 0.12*

^a^Sera at 2 h and 9 h after LPS injection were collected for cytokines assay. The cytokine production in serum was assayed by ELISA kits. Values are means ± SD. ^#^0.05 < *P* < 0.1, *P < 0.05, or **P < 0.01, significantly different from the control group analyzed by Student's *t*-test.

**Table 2:** Chemical compositions of SOE analyzed by GC-MS.

No.	Component	Rt (min)^a^	R. match	Percentage (%)^b^
1	2-Oxabicyclo[2,2,2]octane-6-ol	20.72	763	1.8
2	2-tert-Butyl-1,4-dimethoxy-benzene	23.13	805	3.8
3	Caryophyllene	23.59	866	3.1
4	*cis*-*α*-Bisabolene	26.57	860	4.1
5	[−]-Spathulenol	27.62	853	25.7
6	Caryophyllene oxide	27.95	858	46.9
7	*cis*-Lanceol	29.28	719	1.7
8	[Z,Z,Z]-9,12,15-Octadecatrienoic acid ethyl ester	32.49	736	1.2
9	6,10,14-Trimethyl-2-pentadecanone	33.53	790	2.1
10	Hexadecanoic acid ethyl ester	36.93	794	9.6

^a^Retention time (min).

^b^Relative percentage calculated by integrated peak area.
