# Supplementary material for: Anti-Inflammatory Effects of Siegesbeckia orientalis Ethanol Extract in In Vitro and In Vivo Models
Source: Biomed Res Int. 2014 Aug 26;2014:329712. doi: 10.1155/2014/329712 (PMC4160630; doi:10.1155/2014/329712)
Supplement: Supplementary file 1 — The Siegesbeckia orientalis L. samples were purchased from Yuanshan Company. The sample's original and its DNA polymorphism had been identified. The samples were extracted with 95% ethanol into the extracts (SOE). The reagents lipopolysaccharide and λ-carrageenan were for inflammation induction; Indomethacin and ammonium pyrrolidinedithiocarbamate were as anti-inflammatory reagents. [file 329712.f1.pdf]

Supplementary Table

Chemical compositions of SOE analyzed by GC-MS.

No.	Component	Rt (min)[a]	R. match	Percentage (%)[b]
1	2-Oxabicyclo[2,2,2]octane-6-ol	20.72	763	1.8
2	2-tert-Butyl-1,4-dimethoxy-benzene	23.13	805	3.8
3	Caryophyllene	23.59	866	3.1
4	*cis*-α-Bisabolene	26.57	860	4.1
5	[-]-Spathulenol	27.62	853	25.7
6	Caryophyllene oxide	27.95	858	46.9
7	*cis*-Lanceol	29.28	719	1.7
8	[Z,Z,Z]-9,12,15-Octadecatrienoic acid ethyl ester	32.49	736	1.2
9	6,10,14-Trimethyl-2-pentadecanone	33.53	790	2.1
10	Hexadecanoic acid ethyl ester	36.93	794	9.6

[a]Retention time (min)

[b]Relative percentage calculated by integrated peak area
